# Pancreatic acinar cell carcinoma with extension into the main pancreatic duct: a case report

**DOI:** 10.1186/s40792-021-01172-0

**Published:** 2021-04-13

**Authors:** Masato Kayahara, Ichiro Onishi, Naoki Makita, Shunsuke Kano, Masayoshi Munemoto, Yasumichi Yagi, Makiko Minami, Noriaki Orita, Takuya Komura, Nozomu Kurose

**Affiliations:** 1 Department of Surgery, NHO Kanazawa Medical Center, 1-1 Shimoishibikicho, Kanazawa, Ishikawa 920-8650 Japan; 2 Department of Radiology, NHO Kanazawa Medical Center, 1-1 Shimoishibikicho, Kanazawa, Ishikawa 920-8650 Japan; 3 Department of Gastroenterology, NHO Kanazawa Medical Center, 1-1 Shimoishibikicho, Kanazawa, Ishikawa 920-8650 Japan; 4 Department of Pathology, NHO Kanazawa Medical Center, 1-1 Shimoishibikicho, Kanazawa, Ishikawa 920-8650 Japan

**Keywords:** Pancreatic acinar cell carcinoma, Main pancreatic duct extension, EUS-FNA

## Abstract

**Background:**

Pancreatic acinar cell carcinoma (PACC) is a rare exocrine malignant tumor. Its widespread intraductal extension into the main pancreatic duct (MPD) is also rare.

**Case presentation:**

We report the case of a 71-year-old man with PACC with MPD extension. The patient was assessed with laboratory and radiographic investigations that facilitated a preoperative diagnosis. Endoscopic ultrasonography (EUS) and dynamic thin-slice multi-detector row computed tomography (MDCT) were useful for determining the resection line of the pancreas. EUS-guided fine needle aspiration (EUS-FNA) was also helpful in determining the tumor biology and treatment strategy. Distal pancreatectomy was performed. The MPD was occupied by the tumor 35 mm downstream and 5 mm upstream. Histopathologically, the pancreatic tail tumor extended continuously into the MPD. The tumor was solid with cells showing eosinophilic and granular cytoplasm, indicating the diagnosis of PACC. This is an interesting case of PACC with intraductal extension into the MPD. We discuss the possible mechanisms of tumor extension in this rare case together with a review of the literature.

**Conclusions:**

We describe a rare pancreatic acinar cell carcinoma that could be adequately treated using preoperative precise imaging and histopathological evaluations. When an intraductal tumor extension in the MPD is encountered, the diagnosis of a rare pancreatic tumor should be considered, as in our case.

## Background

Pancreatic acinar cell carcinoma (PACC) is a rare exocrine malignancy with an incidence of less than 1% of all pancreatic neoplasms [1]. The diagnostic methods for PACC include ultrasonography (US), endoscopic ultrasonography (EUS), computed tomography (CT), and magnetic resonance imaging (MRI). Recently, EUS-guided fine needle aspiration (EUS-FNA) has been widely advocated as a standard method for the histopathological diagnosis of a pancreatic mass. Although EUS-FNA is a highly effective diagnostic tool, the preoperative diagnosis of PACC is rare.

We recently treated a patient with PACC that could be preoperatively diagnosed using EUS-FNA. Pathological findings from the resected specimen revealed an interesting mode of tumor spread. In this paper, we report a case of PACC with widespread intraductal extension of the main pancreatic duct (MPD) and relate our findings to the literature.

## Case presentation

A 71-year-old man was admitted to the hospital for strict glycemic control in June 2020. He had been treated for diabetes mellitus (DM), hepatic hemochromatosis, and bronchial asthma for 15 years. Previously, he had undergone a left adrenalectomy for adrenal neuroma. His glycemic control had deteriorated since April. A pancreatic tumor was identified by US, enhanced CT, and MRI. At the request of his family, he was referred to our hospital in August 2020.

On physical examination, no tumor mass was palpable in his abdomen, except for surgical scars. The results of the laboratory investigations were as follows: RBC, 4.5 × 106/μ; hemoglobin, 9.6 g/dl (normal range: 13.0–17.6); WBC, 3.1 × 103/μ (4.5–9.0 × 103); platelet count, 195 × 103/μ; Fe, 23 μg/dl (49–219); amylase, 89 U/l; trypsin, 1171 ng/ml (100–550); elastase-1, 959 ng/dl (0–300). The results of the liver function tests were within normal limits. The levels of tumor markers were as follows: carcinoembryonic antigen (CEA), 3.0 ng/ml; carbohydrate antigen 19-9 (CA19-9), 20 U/ml; Span-1, 39 U/ml (< 30); NCC-ST-439, 1.2 U/ml (< 4.5); AFP, 2 ng/ml. His pulmonary function was impaired, as shown by the following results: FVC, 3.7 l; FEV1, 1.61 l; FEV1%, 43.5%.

**Fig. 1:**
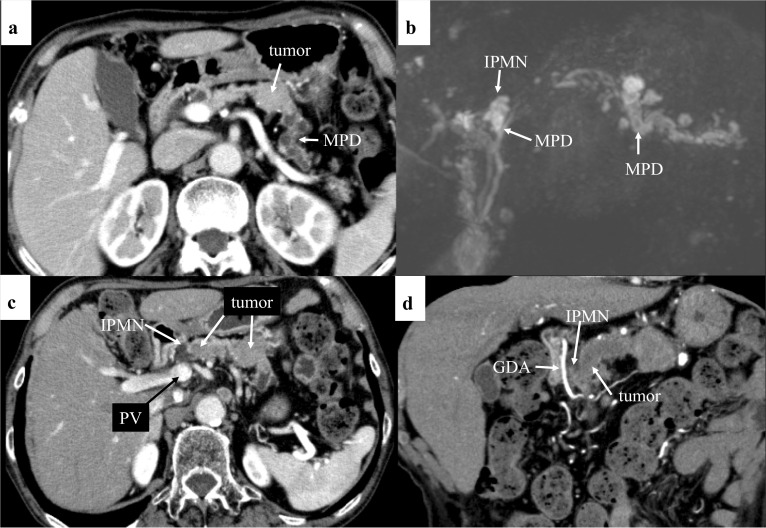
**a** Multi-detector row computed tomography (MDCT) shows a slightly enhanced round tumor and the dilated caudal main pancreatic duct (MPD). **b** Magnetic resonance cholangiopancreatography (MRCP) demonstrates mild dilatation of cranial MPD and severe dilatation of caudal MPD. Cystic lesion [suspicious intraductal papillary mucinous neoplasm (IPMN)] is visible in the neck of the pancreas. **c** MDCT shows a tumor with downstream progression until the left side of IPMN. **d** A coronal view shows intraductal tumor mass extends to the left side of the IPMN

The CT scan showed a slightly enhanced mass, approximately 27 mm in size, in the tail of the pancreas, with dilatation of the distal MPD (Fig. 1a). Magnetic resonance cholangiopancreatography (MRCP) showed mild dilatation (3.5 mm) of the caudal MPD up to the pancreas neck. The distal MPD was dilated to a diameter of 7 mm. In addition, a cystic lesion, a suspected branch duct intraductal papillary mucinous neoplasm (BD-IPMN), was seen in the pancreatic neck (Fig. 1b).

Thin-slice multi-detector row computed tomography (MDCT) in our hospital showed a homogenous slightly enhanced mass with expanded extrapancreatic progression and moderate dilatation of the distal MPD. However, this tumor showed no infiltration pattern to the retropancreatic tissue and splenic vein. Downstream tumor progression in the MPD was observed up to the left side of the portal vein (Fig. 1c, d). These preoperative findings support a diagnosis of PACC or pancreatic neuroendocrine tumor (pNET) rather than pancreatic ductal adenocarcinoma (PDAC).

**Fig. 2:**
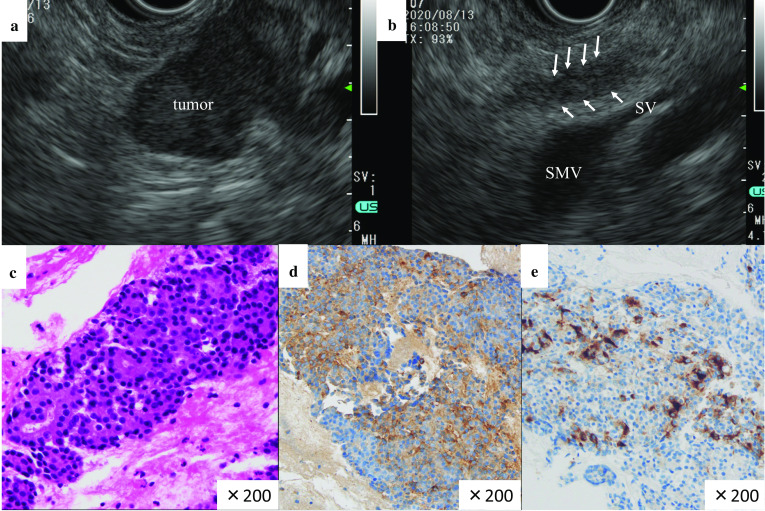
**a** Endoscopic ultrasonography (EUS) shows a low-echoic solid mass in the pancreatic tail. **b** EUS shows intraductal tumor infiltration in the MPD. The splenic vein is intact. White arrows show the downstream intraductal infiltration of tumor in the MPD. **c** Endoscopic biopsy specimen for hematoxylin and eosin (H&E) staining reveals an acinar growth of tumor cells with eosinophilic and granular cytoplasm (×200). **d** Immunohistochemistry shows passivity for α1-antitrypsin (×200). **e** Immunohistochemistry shows positivity for B-cell lymphoma/leukemia 10 (×200)

**Fig. 3:**
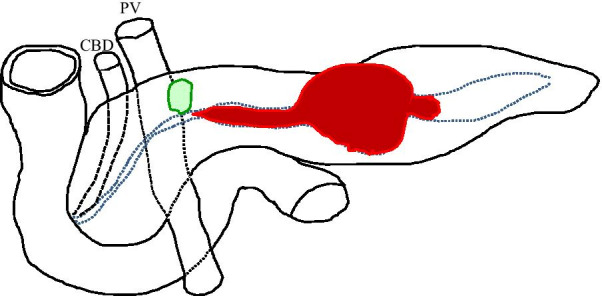
The scheme illustrates the tumor progression based on the preoperative radiographic examinations. The blue dashed line indicates the MPD. The red color represents the tumor. The green line indicates the pancreatic cyst

The EUS findings were as follows: a hypoechoic mass lesion with a lobulated structure approximately 3 cm in size was found in the tail of the pancreas; dilatation of the upstream MPD and a tumor thrombus in the MPD downstream from the main tumor was observed (Fig. 2a, b). EUS-FNA using a 22G needle was performed to obtain specimens for pathological diagnosis. Macroscopic inspection showed whitish fragments. Hematoxylin–eosin (HE) staining showed suspected PACC. The results of the immunohistochemical staining were as follows: chromogranin A (−), synaptophysin (−), CD56 (−), CK7 (+), CK19 (−), β-catenin in the nucleus (−), MIB-1 (20–30%), α1-antitrypsin (+), and B-cell lymphoma/leukemia 10 (BCL-10) (+) (Fig. 2c–e). Based on these results, the pancreatic mass was preoperatively diagnosed as PACC. Figure 3 shows a schematic diagram illustrating the tumor extension based on preoperative imaging.

Considering the preoperative diagnostic evaluations and the patient’s poor physical condition, we scheduled a distal pancreatectomy with a pancreatic dissection line was at the right side of the SMV. A laparotomy was performed. No metastatic lesions were found in the peritoneum or liver. Intraoperative cytological examination was negative for malignant cells. There was no evidence of tumor invasion of the portal venous system or retropancreatic tissue. Distal pancreatectomy with lymphadenectomy in the area of the common hepatic and celiac arteries was performed. The pancreatic neck was transected at the side of the SMV. The postoperative course was uneventful. We presented adjuvant chemotherapy with S1, but the patient refused. There has been no relapse for 5 months postoperative follow-up.

**Fig. 4:**
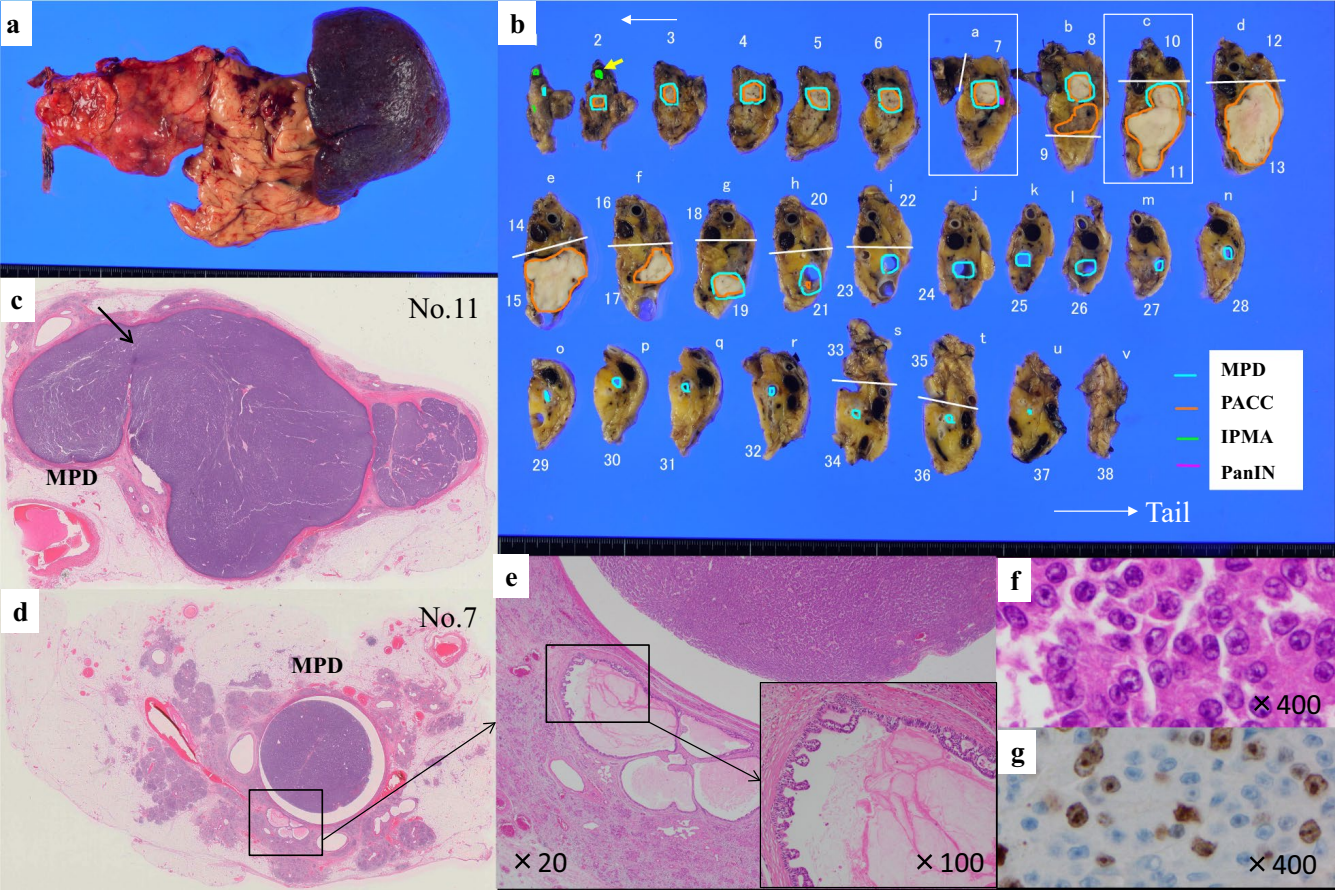
**a** Macroscopic fresh view of the resected specimen showing the main tumor with a rough surface seen in the tail of the pancreas; the macroscopic abnormalities are not recognized. **b** Macroscopic view of the cut surface of the resected pancreas’ body and tail showing the tumor marked in the red circle. The blue circle indicates MPD. Small intraductal papillary mucinous adenoma (IPMA) is visible in the neck of the pancreas (green). Yellow arrow indicates IPMA. Pancreatic intraepithelial neoplasia-3 (PanIN-3) cells are found near the MPD of the pancreatic body (pink), presented in **d**, **e**. **c** Loupe imaging of the pancreatic tail shows the tumor replacing acinar lobules and occupying the MPD. Tumor cells penetrate the MPD (arrows). **d** Tumor mass is noted in the center of MPD. Tumor cells do not invade into the MPD. PanIN-3 cells are in the branched pancreatic ducts near the MPD (see **e**). **e** H&E staining of PanIN-3 around the MPD. Carcinomas in site lesions are found in a section of the peripheral pancreatic duct (×100). **f** H&E staining reveals an acinar growth of tumor cells with eosinophilic and granular cytoplasm (H&E × 400). **g** This figure illustrates Ki67 immunohistochemistry from this patient. MIB1 index was 20–30% (×400)

On macroscopic appearance, the tumor was well-circumscribed, and its cut surface showed a solid tumor. The surgical specimen measured 6.8 × 3.3 × 2.3 cm including the MPD tumor extension. The size of the main tumor, except for the MPD extension, was 3.3 × 2.3 × 2.5 cm. The MPD was occupied by the tumor spreading 35 mm to the duodenum (downstream) and 5 mm upstream. The tumor was gray-white and firm. In macroscopic views of the cut surface, continuous tumor extension into the MPD was observed in the pancreatic body (Fig. 4a, b).

Histologically, the solid mass confirmed the PACC diagnosis suspected by the intraductal growth and preoperative evaluation. No tumor infiltration was observed in the epithelium of the main pancreatic duct (Fig. 4c). A tiny main duct intraductal papillary mucinous adenoma (MD-IPMA) was also observed. No vascular or neural invasion was observed. No cancer cells extended to the splenic vein nor was there nodal involvement. There were no cancer cells at the surgical margins. PanIN-3 cells were seen in the branched pancreatic ducts near the MPD (Fig. 4d, e).

Microscopically, the tumor consisted of small round cells with occasional acinar patterns (Fig. 4f). The results of immunohistochemical staining of the resected specimen were as follows: chromogranin A (−), CA19-9 (−), synaptophysin (−), CD56 (−), p53 (< 5%), maspin (−), α1-antitrypsin (+ weak), and MIB-1 (30%, hot spot) (Fig. 4g). The final diagnosis was T3, N0, M0, Stage IIA (Union for International Cancer Control Staging, 8th edition).

## Discussion

We report a case of PACC with widespread extension of the MPD. Wisnoski et al. [1] reported that 16% of PACC cases had localized disease, 26% had regional disease, and 58% had distant metastases, compared with 10%, 33%, and 57% of patients with PDAC, respectively. Patients with PACC were more likely to have tumors at the body/tail of the pancreas [1, 2]. Patients with PACC have been reported to be predominantly male in the fifth or sixth decade, younger than those with PDAC [1–6]. Several papers using big data analysis showed that the prognosis of PACC was better than that of PDAC [1, 3, 7]. Ban et al. [5] also reported that the respective 5-year survival rate for patients with PACC was 85.7%. A recent report [6] has shown that the prognosis of PACC is extremely good, with a median survival of 105 months in non-metastatic cases. To sum up the findings on PACC prognosis, patients with PACC have better survival rates compared to those with PDAC when a curative resection can be achieved.

Radiographically, PACC is usually a large, oval, exophytic, and well-margined mass. PACC compresses the pancreatic ducts as pNETs and may not cause MPD dilatation [8]. In our case, imaging examinations revealed a relatively well-defined pancreatic tail tumor with caudal MPD dilatation. Therefore, although this tumor was diagnosed as a malignant pancreatic tumor, the pattern of tumor staining was different from the typical PDAC with desmoplastic response. Our patient had mild cranial MPD (3.5 mm) dilatation and moderate caudal dilatation (7 mm). PDAC derived from IPMN needs to be ruled out.

The histological findings of PACC may sometimes mimic pNET. EUS and EUS-FNA play important roles in the diagnosis of pancreatic tumor [9]. Seeding of a pancreatic tumor to the gastric wall through the biopsy needle tract or peritoneal dissemination are uncommon but has been reported in some cases [10]. The use of EUS-FNA in the diagnosis of pancreatic cancer has been found to not increase the risk of peritoneal carcinomatosis significantly [11]. Our current diagnostic strategy for a pancreatic mass is to obtain preoperative histological diagnosis using EUS-FNA. Recently, our group reported that EUS-FNA provided important information for a differential diagnosis in a patient with a lymphoepithelial pancreatic cyst [12]. To the best of our knowledge, only one case [13] with a preoperative histological diagnosis and radical resection, which had MPD extension, has been reported in the English literature.

Intraductal growth of PACC is rare. PACC with MPD involvement was first reported in 2001 [14]. Hashimoto et al. [15] reported that intraductal spreading tumors with lymphatic, venous, and neural invasion had peripancreatic nodal involvement. At that time, the pathological features of this tumor were not yet known. Bhosale et al. [16] reported that only 10% of PACC cases had pancreatic ductal ingrowth, which showed a papillary pattern similar to that of IPMN [17]. Ban et al. [5] reported the gross and histological features of 13 cases of PACC, of which 7 showed intraductal polypoid growth (IPG). These authors described the macroscopic appearance of the tumor as a unique gross shape, which was referred to as “sausage-like”. However, the main tumor in our case was oval, and the distal pancreas was atrophic. They also mentioned that only 1 of 7 cases of PACC with IPG showed lymphatic invasion, but 5 of the 6 tumors without IPG showed lymph-vascular or neural invasion. Our case had no nodal, lymph-vascular or neural involvement. Although the size of the main tumor was approximately 3 cm in diameter, the tumor extension in the MPD was 35 mm or more. This indicates that the main tumor can invade the pancreatic duct with relative ease and expand into the MPD.

PACC with an intraductal tumor spread might be less invasive to the lymphatic or venous root as mentioned above. We suggest the following possible mechanisms for this phenomenon: (1) PACC is a medullary tumor and is less invasive compared to PDAC and extends widely within the pancreatic duct epithelium; (2) the downstream MPD pressure is lower than the upstream pressure; (3) PACC extends into pancreatic ducts when it is a relatively small mass and the pancreatic ducts are ruptured near the MPD. In summary, this tumor can easily spread from the caudal side to the duodenal side. This mechanism may be similar to that of tumor thrombus formation in the portal system [13].

Radical resection is the most important prognostic factor. Among the locoregional pancreatic cancers, PACC has a higher curability rate than PDAC [1]. The question of the evaluation of curability and postoperative quality of life remains. Total pancreatectomy (TP), which might cause cholangitis, brittle diabetes, and poor nutrition, should be considered depending on the tumor extension in the MPD. Whether or not we select TP is a difficult issue for patients with poor physical condition, as in our case. The tumor extension in our case was determined not to have spread beyond the left line of the SMV. Ultimately, we decided to perform radical resection as already described. We could obtain complete curability by pathological evaluation.

Recently, neoadjuvant chemotherapy has been recommended for advanced pancreatic cancer. After reduction of the tumor size and extension, surgical treatment may be selected [13]. However, it is unclear whether chemotherapy always results in tumor shrinkage. The effectiveness of postoperative adjuvant therapy with PACC is unclear. Our case could not consent to adjuvant chemotherapy.

## Conclusion

We described a rare pancreatic acinar cell carcinoma that could be adequately treated using precise preoperative imaging and histopathological evaluations. When an intraductal tumor extension in the MPD is encountered, a diagnosis of a rare pancreatic tumor should be considered, as in our case.

## Data Availability

Not applicable.

## Abbreviations

PACC: Pancreatic acinar cell carcinoma
MPD: Main pancreatic duct
US: Ultrasonography
EUS: Endoscopic ultrasonography
CT: Computed tomography
MDCT: Multi-detector row CT
MRI: Magnetic resonance imaging
SMV: Superior mesenteric vein
EUS-FNA: EUS-guided fine needle aspiration
PDAC: Pancreatic ductal adenocarcinoma
pNET: Pancreatic neuroendocrine tumor
IPMN: Intraductal papillary mucinous neoplasm
MRCP: Magnetic resonance cholangiopancreatography
